# Risk Factors of 120-Day Mortality Among Hip Fractures With Concomitant COVID-19 Infection

**DOI:** 10.7759/cureus.32637

**Published:** 2022-12-17

**Authors:** Nuthan Jagadeesh, Venkatesh Deva, Sachindra Kapadi, Debbie Shaw

**Affiliations:** 1 Trauma and Orthopaedics, Wrightington, Wigan and Leigh NHS Foundation Trust, Wigan, GBR; 2 Trauma and Orthopaedics, Royal Infirmary of Edinburgh, Edinburgh, GBR

**Keywords:** coronavirus disease 2019, risk factors of mortality, 120-day mortality, neck of femur fracture, hip fractures, covid-19

## Abstract

Background

Hip fractures cause substantial morbidity and mortality worldwide, and the coronavirus disease 2019 (COVID-19) pandemic has only worsened the global burden. Increased 120-day mortality is well established in the literature among hip fractures with COVID-19. However, the risk factors associated with mortality have been poorly understood. We aimed to determine the risk factors associated with increased 120-day mortality among hip fractures with COVID-19.

Methods

Seventy patients with hip fractures with confirmed COVID-19 infection between March 2020 and December 2021 were included. Thirty-three patients who died within 120 days of admission were allotted to the non-survivor group and the rest 37 patients were allotted to the survivor group. Various parameters such as demographic variables, Nottingham Hip Fracture Score (NHFS), Charlson Comorbidity Index (CCI), American Society of Anesthesiologists (ASA), Abbreviated Mental Score Test (AMTS), National Early Warning Score (NEWS), fracture type, operation type, and delay in surgery were compared between the groups to determine the risk factors for increased mortality. Multivariate regression analysis was performed to know the independent association with increased mortality.

Results

A total of 33 patients died within 120 days giving the 120-day mortality rate of 47.1%. Baseline parameters such as ASA, AMTS on admission, NEWS on admission, and type of residence did not significantly affect mortality. The mean NHFS was significantly high in non-survivors (5.38 ± 1.52) compared to survivors (4.40 ± 1.75) (p < 0.001). Similarly, mean CCI was also significantly high in non-survivors (5.58 ± 1.74) compared to survivors (4.76 ± 2.29) (p < 0.001). A total of 70% (seven out of 10) of patients with delayed surgery of more than 36 hours from the admission died within 120 days of admission (p < 0.001). Mortality was significantly higher in patients who underwent internal fixation of fractures like a dynamic hip screw (DHS) or intramedullary (IM) nailing than in those who underwent hemiarthroplasty or total hip arthroplasty (THA). Post-operative parameters such as early mobilization and the multidisciplinary approach to review these patients made no difference to the mortality. Multivariate regression analysis of the parameters that made a significant difference in the mortality showed that delay in surgery and type of surgery (internal fixation) independently increased the mortality among these patients (p < 0.001). However, NHFS and CCI were not independently affecting the mortality among hip fractures with concomitant COVID-19.

Conclusion

The 120-day mortality rate among patients with hip fractures with concomitant COVID-19 was 47.1%. Delay in surgery of more than 36 hours and patients who underwent internal fixation were independent risk factors associated with increased mortality among these patients.

## Introduction

Hip fractures are among the commonest fracture occurring in the elderly in the United Kingdom (UK). About 63,284 hip fractures occurred in 2020 in the UK and the numbers are predicted to rise [1]. The epidemiology of orthopaedic trauma indicates that there is a reduced incidence of major trauma during the time of coronavirus disease 2019 (COVID-19), but the incidence of fragility fracture remains the same [2-5]. Hip fractures tend to occur in the elderly age group with higher rates of frailty and comorbidity.

According to the National Hip Fracture Database (NHFD) report 2020, the mortality among neck of femur fractures rose to 8.3% compared to 6.5% in 2019 [1]. This meant that over 1,000 more people died during this first year of the COVID-19 pandemic than would have been expected without the COVID-19 pandemic. The NHFD recorded 3,730 patients as having COVID-19 either at the time of their presentation or being diagnosed following admission in the year 2020. For these patients, 30-day mortality was three times higher than seen in those without the infection. The national 30-day mortality among neck of femur fractures with COVID-19 was 20-25%, according to the NHFD report 2020, but numerous studies from individual hospitals in the UK have published mortality rates varying between 30% and 40% [1,6-9]. According to Hall et al., COVID-19-positive patients with hip fractures had a mortality rate of 35.5% after 30 days, and COVID-19 was three times more likely to result in mortality after adjusting for confounding factors, including age, sex, residence, Nottingham Hip Fracture Score (NHFS), and American Society of Anesthesiologists (ASA) grade [10]. In a systematic review by Andritsos et al. evaluating the impact of COVID-19 in the first eight months in the UK, the average 30-day mortality was found to be 37.8% (range: 30.4% to 50%) [11,12]. COVID-19 can spread nosocomially and has a variable incubation period, so patient follow-up should be performed even after 30 days to estimate true mortality. Hip fracture patients have been reported to have a 120-day mortality rate ranging between 12.5% and 20.1% before the pandemic [13-15]. Oputa et al. estimated the 120-day mortality rate to be 63%, which is nearly double the estimated 30-day mortality rate [16]. Estimating the 120-day mortality rate will not only help us to know the true mortality but will also give us the opportunity to assess the risk factors leading to mortality.

The risk factors of increased mortality among hip fractures with COVID-19 are poorly understood. Kayani et al. concluded in a multi-centre study that smoking and multiple comorbidities are the risk factors that increase mortality among these patients [6]. NHFD has also indicated a few key performance indicators, such as the timing of surgery, review by a medical specialist, early mobilization, postoperative delirium, and type of surgery, that influence mortality [1]. With the management of the neck of the femur being a huge financial burden for the National Health Service (NHS) UK [17], it is particularly important to know what factors are causing increased mortality.

This study aimed to determine the risk factors that increase 120-day mortality in hip fractures with concomitant COVID-19 by comparing demographic variables, comorbidities, the timing of surgery, fracture type, operation type, etc. between survivors and non-survivors.

## Materials and methods

This single-centre, retrospective study was conducted in a district general hospital in the UK. All the patients admitted to our hospital between the 1st of March 2020 and the 31st of December 2021 with hip fractures satisfying the inclusion criteria were included. Hip fractures included the fracture of the neck of the femur, intertrochanteric fracture, and subtrochanteric fracture as per the definition by the NHFD. The patient was considered COVID-19 positive if there was documented evidence of the positive results of quantitative reverse transcription-polymerase chain reaction (RT-PCR) and/or clinical and/or radiological findings suggestive of COVID-19 infection. COVID-19 tests were undertaken during admission and at regular intervals during the hospital stay. Patients with open fractures, femoral shaft fractures, distal femur fractures, periprosthetic fractures, and greater trochanter fractures were excluded. The study was registered in the audit and research department of the trust and ethical approval was deemed not necessary due to the retrospective nature of the study by the research department.

Demographic variables like age, sex, ethnicity, and type of residence (care home or own home) were collected from a manual review of patient notes on electronic health records. Data on the type of fracture, intervention, the timing of surgery, and length of hospital stay were collected. NHFS, ASA score, and Charlson Comorbidity Index (CCI) were calculated for each patient. The patients were followed up to 120 days post-admission to estimate the 120-day mortality rate, which is the primary outcome measure. The patients who died within 120 days of admission were included in the non-survivor group and the rest were included in the survivor group. The risk factors causing increased mortality were evaluated by comparing the collected variables between the groups.

Statistical analysis

We descriptively summarized all patient characteristics. For continuous variables, mean and standard deviation were reported. Two categorical variables were analysed using the chi-square (χ2) test. Two independent groups were compared using an unpaired t-test to determine whether their means differed. A p-value of <0.05 indicated statistical significance. Multivariate regression analyses were done to determine the independent influence of each parameter on mortality. To analyse the data, SPSS software v.23 (IBM Corp., Armonk, NY) and Microsoft Office 2007 (Microsoft Corporation, Redmond, WA) were used.

## Results

Seventy patients were diagnosed with COVID-19 during the study period. Thirty-three patients (33 out of 70) among these who died within 120 days of admission were allotted to the non-survivors group giving a 120-day mortality rate of 47.1%. The remaining 37 patients were allotted to the survivor’s group. The mean age and type of fracture diagnosed did not differ significantly between the groups. Baseline parameters such as ASA, Abbreviated Mental Score Test (AMTS) on admission, National Early Warning Score (NEWS) on admission, and type of residence did not significantly affect mortality. The mean NHFS was significantly high in non-survivors (5.38 ± 1.52) compared to survivors (4.40 ± 1.75) (p < 0.001). Similarly, mean CCI was also significantly high in non-survivors (5.58 ± 1.74) compared to survivors (4.76 ± 2.29) (p < 0.001). On looking at individual comorbidities, patients with a past medical history of myocardial infarction (MI), congestive heart failure (CHF), dementia, and chronic kidney disease had higher mortality, but the difference was not statistically significant (p > 0.05). A total of 70% (seven out of 10) of patients with delayed surgery of more than 36 hours from the admission died within 120 days of admission (p < 0.001). All four patients who were treated with non-operative management died but it must be noted that three of these were the patients who were not fit for surgery and the other one died before having surgery. Mortality was significantly higher in patients who underwent internal fixation of fractures such as a dynamic hip screw (DHS) or intramedullary (IM) nailing than those who underwent hemiarthroplasty or total hip arthroplasty (THA). Post-operative parameters such as early mobilization and the multidisciplinary approach to review these patients made no difference to the mortality (Table 1).

**Table 1:** Comparison of various risk factors between the survivor and non-survivor groups.

	Survivors	Non-survivors	Total	P-value
Total no. of patients	37	33	70	
Age in years	79.44 ± 11.14	81.88 ± 10.71	80.69 ± 10.88	0.42
Gender				
Male	12 (44.4%)	15 (55.6%)	27	0.12
Female	25 (58.1%)	18 (41.9%)	43	
Type of fracture				
Neck of femur	21 (56.8%)	16 (43.2%)	37	0.21
Intertrochanteric	16 (53.3%)	14 (46.7%)	30	
Subtrochanteric	0	3 (100.0%)	3	
Nottingham Hip Fracture Score	4.40 ± 1.75	5.38 ± 1.52	4.90 ± 1.70	<0.01
Charlson Comorbidity Index	4.76 ± 2.29	5.58 ± 1.74	5.18 ± 2.05	0.04
Myocardial infarction	1 (14.3%)	6 (85.7%)	7	0.09
Congestive heart failure	2 (22.2%)	7 (77.8%)	9	0.14
Peripheral vascular disease	0	0	0	-
Cerebrovascular accident	5 (45.5%)	6 (54.5%)	11	0.78
Dementia	5 (29.4%)	12 (70.6%)	17	0.07
Chronic obstructive pulmonary disease	5 (35.7%)	9 (64.3%)	14	0.34
Connective tissue disorder	0	0	0	-
Peptic ulcer	0	0	0	-
Liver disease	0	2 (100.0%)	2	-
Diabetes	6 (50.0%)	6 (50.0%)	12	0.93
Hemiplegia	0	0	0	-
Chronic kidney disease	2 (25%)	6 (75%)	3	0.23
Tumour	4 (80.0%)	1 (20.0%)	5	0.19
Acquired immunodeficiency syndrome	0	0	0	-
American Society of Anesthesiologists (ASA)				
I	1 (50.0%)	1 (50.0%)	2	0.84
II	4 (66.7%)	2 (33.3%)	6	
III	28 (53.8%)	24 (46.1%)	52	
IV	4 (37.5%)	6 (62.5%)	10	
Abbreviated Mental Test Score (AMTS)				
<8	8 (36.4%)	14 (63.6%)	22	0.11
≥8	29 (60.4%)	19 (39.6%)	48	
National Early Warning Score (NEWS) on admission				
≤2	29 (53.7%)	25 (46.3%)	54	0.86
>2	8 (50.0%)	8 (50.0%)	16	
Type of residence				
Own home	31 (63.2%)	18 (36.8%)	49	0.11
Care home/nursing home	6 (28.6%)	15 (71.4%)	21	
The time between admission and surgery				
<36 hours	36 (60%)	24 (40%)	60	<0.001
>36 hours	3 (30%)	7 (70.0%)	10	
Type of anaesthesia				
General anaesthesia	5 (25.0%)	8 (75.0%)	13	0.27
Spinal with or without block	32 (54.5%)	21 (45.5%)	53	
Not operated	0	4 (100.0%)	4	
Type of surgery done				
Not operated/conservative	0	4 (100.0%)	4	<0.001
Total hip arthroplasty/hemiarthroplasty	25 (69.5%)	11 (30.5%)	36	
Dynamic hip screw/intramedullary nailing/cannulated cancellous screw	12 (40.0%)	18 (60.0%)	30	
Multidisciplinary team approach				
No	1 (25.0%)	3 (75.0%)	4	0.61
Yes	36 (54.5%)	30 (45.5%)	66	
Next day mobilization				
No	8 (57.1%)	6 (42.9%)	14	0.65
Yes	29 (51.8%)	27 (48.2%)	56	

Multivariate regression analysis of the parameters that made a significant difference in the mortality showed that delay in surgery and type of surgery (internal fixation) independently increased the mortality among these patients (p < 0.001). However, NHFS and CCI were not independently affecting the mortality among hip fractures with concomitant COVID-19 (Table 2).

**Table 2:** Multivariate logistic regression analysis for the risk factors associated with mortality in hip fractures with COVID-19 patients.

	Odds ratio	95% CI	P-value
Nottingham Hip Score	1.35	0.74-2.47	0.32
Charlson Comorbidity Index	1.04	0.62-1.76	0.85
The time between admission and surgery			
<36 hours	Ref		<0.001
>36 hours	7.80	73-82.59	
Type of surgery done			
Total hip arthroplasty/hemiarthroplasty	Ref		<0.001
Dynamic hip screw/intramedullary nailing/cannulated cancellous screw	4.57	1.29-16.18	

## Discussion

Hip fractures cause substantial morbidity and mortality worldwide, and the COVID-19 pandemic has only worsened the global burden. Though the escalated 30-day and 120-day mortality is well established in the literature among hip fractures with COVID-19, the risk factors associated with the mortality have been poorly understood. We aimed to determine the risk factors associated with increased 120-day mortality among hip fractures with COVID-19.

In their study, Oputa et al. reported a 120-day mortality of 63% among hip fracture patients with concomitant COVID-19 [16]. The study period was limited to a month of the initial COVID-19 surge during March 2020, which may not provide the accurate impact of the disease [10]. A better understanding of the disease and judicious use of resources with COVID-19 vaccination has bought a sense of control of the disease. However, in our study covering two years since the pandemic, the overall 120-day mortality is still high at 47.1% (33 out of 70). This only substantiates the need to evaluate the risk factors increasing mortality among these patients.

In the multicentric study evaluating inpatients across the UK, Docherty et al. suggested that males were not only more likely to be infected by COVID-19 but are also at risk of increased mortality [18]. In the IMPACT-Scot study, Hall et al. suggested that males with hip fractures were at increased risk of mortality compared to females with concomitant COVID-19 [10]. Similarly, Oputa et al. also reported that males are at higher risk of mortality [16]. In our study, though males had higher mortality than females, the difference was not statistically significant (p = 0.12).

When infected with COVID-19, patients with hip fractures are likely to experience complications due to their frailty and multiple comorbidities. Kayani et al. reported that patients with more than three comorbidities were at increased risk of mortality [6]. Hall et al. also reported that NHFS of >7 was a significant risk factor for increased mortality [10]. In our study, both mean NHFS and CCI were significantly high among the non-survivors compared to the survivors. Amongst the comorbidities, both Hall et al. and Oputa et al. suggested pulmonary disease increased the risk of mortality in these patients [10,16]. In our study, patients with a history of MI, dementia, and chronic kidney disease had higher mortality but the difference was statistically significant (p < 0.001). Chronic obstructive pulmonary disease made no difference to the mortality (p > 0.05).

Best practice guidelines recommend that patients with hip fractures be operated on within 36 hours to decrease mortality. Dar et al. suggested that having delayed surgery did not significantly increase mortality [19]. The findings of this study are quite contradictory to a systematic review done by Khan et al., who suggested early surgery for hip fractures decreases both the mortality and length of hospital stay [20]. In our study, 70% of patients who had delayed surgery of more than 36 hours died within 120 days of surgery (p < 0.001). Even multivariate regression analysis also suggested a delay in surgery independently increased mortality among these patients. This substantiates the need for early surgery to decrease mortality. Other key performance indicators like early mobilization and medical specialist input did not significantly improve the mortality in our study.

Kayani et al. found no difference in mortality based on the type of surgery, i.e., internal fixation or hemiarthroplasty [6]. Similar results were also found in the study by Hall et al. [10]. However, in our study, patients who underwent internal fixation had significantly higher mortality compared to those who underwent hemiarthroplasty or THA. Multivariate regression analysis also suggested patients who had internal fixation had increased mortality among these patients. Though the type of fracture made no difference, all three patients with subtrochanteric fractures who underwent intramedullary nailing died within 120 days of surgery.

Several limitations have been identified in this study. Compared to multicentric studies, this is a single-centre study with a relatively small number of patients. The study does not account for the influence of staff redeployment, changes in hospital policies through the pandemic, lockdowns, and COVID-19 vaccination on mortality. However, this is the first study that evaluates risk factors for 120-day mortality and the knowledge we gain from this study can help us develop better hospital policies to decrease mortality rates.

## Conclusions

The 120-day mortality rate among patients with hip fractures with concomitant COVID-19 was 47.1%. Delay in surgery of more than 36 hours and patients who underwent internal fixation were independent risk factors associated with increased mortality among these patients. The influence of COVID-19 vaccination and changes in hospital policies on mortality need to be evaluated in future studies.
